# Elephantiasis Græcorum and Elephantiasis Arabum

**Published:** 1829-10-01

**Authors:** 


### XXVIII.

### ELEPHANTIASIS GRÆCORUM; AND CURIOUS AFFECTION OF THE LOWER EXTREMITY, RESEMBLING ELEPHANTIASIS ARABUM.*

The first case to which we shall advert is contained in a memoir on the Elephantiasis Græcorum, published by M. Cazenave in the Journal Hebdomadaire.

M. ——, 40 years of age, unmarried, had resided for 20 years in the French colonial islands, without ever having experienced ill health. In 1822 he contracted the itch, which was cured in the course of a few days by lotions of the "Eau de Mettemberg," and shortly afterwards he became affected with a gonorrhœa, for which he used no treatment. He had laboured under the latter fifteen days, when suddenly he found upon his cheeks and thighs a number of tubercles, as large as small nuts, prominent, irregular, red, and extremely sensible to the touch. Fifteen days after the appearance of this eruption he lost his clap, and the whole surface of the skin now became so exquisitely tender, that the slightest touch on any part produced a sensation similar to that which a blow upon the ulnar nerve at the elbow produces. By means of baths and a drink of sarsaparilla, the tubercles, and all the other features of the patient's complaint, were completely dispersed in the course of two months. It may be right to mention that, previous to the present attack, M. —— had lived almost exclusively on salted pork.

A year had elapsed, and the patient perfectly retained his health, when, unfortunately for himself, he set off to attend a fête ten leagues from home. He drank there a quantity of warm wine, fatigued himself greatly, and returned on foot in the midst of "a pour of rain." He went to bed extremely ill, and next day the tubercular eruption had re-appeared. Its progress was this time frightfully rapid, for in very few days it had invaded, not merely the thighs, but the ears and the whole face, whilst the eye-brows and eye-lashes had completely fallen off. A variety of medicines were employed in vain, and amongst other means, are enumerated *lizards*, which he swallowed alive! Four years passed away, but, instead of getting better, the malady got worse; the tubercles increased in number; the red colour gave way to a bronzed tint; and, finally, his sight began to fail him, when he made up his mind to repair from the Isle of France, where he was, to Paris, and entered the Hôpital St. Louis, on the 22d of August, 1827, under the care of M. Biett.

The face was of shining bronze colour, unequal on its surface, bloated, and covered with a crowd of tubercles, separated from one another by sulci, varying in depth. These tubercles were nearly as large as nuts, soft, and almost insensible. The ears were deformed; the lips enlarged and nodulated; the mucous membrane of the mouth of a bronze colour: the eye-brows and eyelids gone; the inferior part of the forehead deeply grooved, whilst a large soft tubercle between each groove, hanging down before the eye, answered excellently well to the Greek designation of the *leontine disease*; the transparent corneæ surrounded by a circle like that in chemosis, but of bronze hue; the iris of the left eye adhering to the pupil, which was not sufficiently large to admit the introduction of a pin's head. The tubercles in the extremities were larger and less prominent than those on the face, and the subcutaneous cellular membrane was swoln and flabby. The prepuce was affected in a similar way, but the trunk, though partaking of the peculiar colour observed elsewhere, was otherwise free from the disease. There were constant attacks of diarrhœa rapidly succeeding one another, and tardily yielding to opiates and blisters to the ileo-cæcal region. Several slight attacks of erysipelas visited the face during his stay at the St. Louis, but this made very little or no alteration in the state of the complaint. On the 10th of November the patient left the hospital, no better than when he entered, the diarrhœa

* Journ. Hebdomadaire, Nos. 30-33.

having prevented the employment of any remedies directed to the elephantiasis. The man, it should be noticed, had not the least sexual desire.

In the course of six months, M. —— returned to the St. Louis in a still worse state than before. The hands, which had been but slightly affected, were now very much so; the sight was almost totally destroyed; the voice was raucous and nearly inaudible; but the alimentary canal was improved in its condition, and M. Biett determined on the exhibition of iodine. Five or ten drops of the tincture were given at first, and the dose augmented by degrees to twenty in a five-ounce mixture, whilst frictions with the ioduret of sulphur and lard, and vapour-baths were ordered in addition. During this treatment, which was pursued for the space of two months, there were more attacks of erysipelas of the face, one of them very severe, and, more than once, the body became covered by small pustulous vesicles, which were spontaneously re-absorbed. Finally, there appeared large bullæ on the legs, and especially the knees, which looked as if the patient had been burnt, but were proved, by direct experiment, to depend upon the iodine. The patient appeared, on the whole, to be improving, when violent diarrhœa again supervened, and continued for three months, in spite of every endeavour to arrest it. Cough succeeded, and bloody expectoration, which, with other symptoms, and the indications furnished by the stethoscope, shewed the existence of pneumonia, under which the patient sank, on the 21st of March, 1829.

*Sectio Cadaveris.* The discoloration of the skin was singularly diminished, and, what is suprising, the tubercles had almost entirely disappeared. In the limbs, not only these, but the swelling were gone, and only a little dulness of tint remained, but the face was still preternaturally soft, though the insulated tubercles were gone. After maceration of the skin for several days, the epidermis was found, on dissection, to be thickened, and beneath it was an extremely vascular, and, indeed, erectile tissue, the density of which was greatest the farther from the surface, and of bronze-like colour. Beneath this was another tissue, hard, dense, bronzed and solid, presenting several little cavities, containing transparent, or yellowish white concretions. Beneath this, again, was a thick adipo-cellular texture.

The brain was rather firm, a little injected at its surface, and presenting a slight congestion of the vessels at its base. The spinal marrow was in a similar state.

The larynx and bronchi were full of thick mucus. The internal membrane of the former was of bronze colour and unequal granular surface, besides which it was thickened, ulcerated in some parts, tumefied in others, and softened throughout. This alteration was particularly remarkable in the inferior chordæ vocales, in the sacculi laryngis, and inferior surface of the epiglottis, which was somewhat enlarged. The cellular membrane external to the mucous was thickened. The mucous membrane of the trachea and bronchi was red, soft, and thickened, which latter was the case, to a remarkable degree, with the external cellular tissue. Following the ramifications of the bronchi, the hypertrophy of all the tissues increased—a quantity of bloody fluid was effused into the pleuræ—and the characteristic marks of peripneumony were presented in the lungs, but not a tubercle in any point.

Some yellow serum existed in the pericardium, and the lining membrane of the great vessels was stained of vinous red colour by the blood, which was universally fluid.

The mucous membrane of the alimentary canal, especially of the stomach and large intestine, was of bronzed colour. The follicles at the base of the tongue and in the stomach were greatly developed, but unmixed with tubercular growths. At the great end of the stomach the mucous membrane was softened, at the pyloric it was the reverse—there were several ulcerations in the colon.

The kidneys, spleen, liver, were soft, voluminous, and gorged with blood, but free from any tubercles.

The optic nerves were sound—the conjunctiva on the eye-ball and the eye-lid was thickened and deeply bronzed. The laminæ of the corneæ were thick and yellow; the aqueous humour scant and turbid; the capsule of the right lens opaque, but the lens itself quite healthy, whilst the contrary had taken place on the opposite side; the hyaloid membrane on both sides thick and opaque, and the vitreous humour itself rather muddy on the right; and, finally, the retinæ were sound.

The testicles, the epididymes especially, the glans penis and the prepuce, were changed into a resisting, hard, dense, and lardaceous tissue, which crepitated when cut. The corpora cavernosa were exsanguined, and their fibrous septa enlarged. The bronzed yellow tint existed almost every where, even in the bones, but the muscles were exempt and of bright red hue.

We have given the dissection thus minutely, because it is that of a disease whose rarity precludes our having many opportunities of learning its pathological characters, at least in this country. We fear, however, that here, as in all the diseases of the skin, morbid anatomy is of very little service. Not only does it lead to no sort of practical result, but it does not even throw any accurate light on the anatomical nature of the malady, at least so it seems, perhaps erroneously, to us. The history of the case and effects of the medicines prescribed, will be read with more interest by the English practitioner, than the notes of a patient and laborious dissection, because they are matters with which he may chance to be professionally interested. Therapeutics are pound, shillings and pence affairs, and, therefore, incomparably more important to John Bull than morbid anatomy, which is looked upon by many as a mere scientific bug-bear! The disappearance of the disease, then, under the use of sarsaparilla, its sudden re-appearance after the fatigue and exposure to wet, and, lastly, the effects of iodine, are not unworthy of remark. The organic alteration of the genital organs satisfactorily accounts for the impotence during life, a fact which has given rise to much dispute amongst the writers on elephantiasis. Altogether the case is well worthy a place in our columns, as well as the attention of our readers, and we assure the latter, that the memoir from which it is extracted is much more bulky than the form in which it is presented here.

The next case occurred at Leipsic, in the Hospital Saint Jacob, under the care of Professor Clark, and is detailed in a thesis published in that city, by M. R. F. A. Martini. Its history presents sufficient points of singularity to make it deserving of notice.

The patient was a weaver, Charles Hackell by name, ætatis 40, of bilious temperament, and father of five healthy children. When eight years of age, he was attacked, for the first time, with severe erysipelas of the right foot, which terminated in vesications, and then left the part in its natural state. In the course of a fortnight, a second attack of the same disease occurred in the same spot, and from this time till the fourteenth year of the patient's life, he had many such, at intervals of longer or shorter duration. At this time no change in the size of the foot had taken place, and Hackell could walk as well with that as with the other. Subsequently, however, each successive attack of erysipelas left a slight deposit behind it, till at length the new matter acquired a remarkable thickness.

The foot and leg are at present exactly the shape of the elephant's. The enlargement commences at the middle of the tibia, above which point the skin is glossy and of bright red colour, and dispersed over its surface are tubercles, the size of a pea, some of them as large as a small nut. The cellular tissue is perceptibly indurated, and gradually blends itself with a thick layer of adventitious scaly matter, fissured here and there, of a dirty earth colour, and resembling the crust of bread. This morbid deposit extends on the dorsum of the foot to the very extremities of the toes, and the deep fissures at every joint, together with the excessive enlargement at all other points, combine to produce a very hideous appearance. The sole of the foot is entirely free from the deposit, and at several points on its sides, the skin of the former is separated from it by a white line. The structure of the crust seems to consist of an aggregation of small processes, closely drawn together and dry upon their surface. It is evident, says the narrator, that they spring from the epidermis, because they can be separated from the subjacent parts without the least pain or a drop of blood. The patient experiences no pain in the part, nor any inconvenience, except from the weight of the foot. He is perfectly sensible to the least impression, or even the slightest tickling on the foot. Amongst all the means that have been tried in the treatment of the disease, compression alone has been productive of advantage. The frequency, however, of the return of erysipelas, and the severity of the pains attending it, having increased *pari passu* with the diminution of volume in the leg, the patient has been compelled to desist from bandaging, and at present employs no treatment whatever.

The author of the case, M. Martini, considers the case as one of erysipelas and nothing else, and denies its identity with elephantiasis. That it is not such a case as that which is first detailed in the present article, the tuberculous disease of the face, &c. in fact the elephantiasis of the Greeks, is very clear; but, as the reviewer in the Journal Hebdomadaire remarks, it is certainly an instance of the elephantiasis Arabum, or elephantiasis in the proper sense of the term. The reviewer in question, M. Cazenave, has seen it succeed the cicatrization of old ulcers at the Hôpital St. Louis, and Dr. Bouillaud has witnessed it after the obliteration of veins. That these were local causes for the disease is highly probable, but whether the erysipelas stood in the same relation to the complaint in the present instance, or was merely a symptom attendant upon it, would be very difficult indeed to determine.

It appears, from a notice in the Hebdomadaire, that another case of the disease has been published by M. Hosack, in a Thesis at Leipzic, like the former. We have not the details at hand, and if we had, our limits would scarcely allow our making use of them, for

What so tedious as a thrice-told tale?

